# Causal links to missed Australian midwifery care: What is the evidence?

**DOI:** 10.18332/ejm/127769

**Published:** 2020-10-29

**Authors:** Ian Blackman, Eleni Hadjigeorgiou, Liz McNeill

**Affiliations:** 1 College of Nursing and Health Sciences, Flinders University, Marion, Australia; 2 Nursing Department, Faculty of Health Sciences, Cyprus University of Technology, Limassol, Cyprus

**Keywords:** modelling, Australia, missed midwifery care

## Abstract

**INTRODUCTION:**

The incidences and types of missed nursing care in the acute care and community sectors are both ubiquitous and quantifiable, however, there are few research studies relating to the type and frequency of missed maternity-based care for mothers and families. The aim of this study is to estimate the incidences and types of Australian missed midwifery care and to identify those factors that have causal links to it.

**METHODS:**

A non-experimental, descriptive method using a Likert developed MISSCARE scale was used to ascertain consensus estimates made by Australian midwives. Electronic invitations were extended to their membership using an inclusive link to the MISSCARE survey. Inclusion criteria were all ANMF members who were midwives and currently employed within the Australian public and private healthcare systems. Data analysis was undertaken using both Rasch analysis and Structural Equation Modelling.

**RESULTS:**

The type and frequency of missed Australian midwifery care can be quantified and several demographic factors are significant predictor variables for overall missed midwifery care. The most prevalent aspects of missed care in the Australian midwifery setting are midwives’ hand hygiene, supportive care, perinatal education, and surveillance type midwifery practices.

**CONCLUSIONS:**

As the frequencies and types of missed midwifery care in Australia have been identified, it is possible for midwives to be mindful of minimising care omissions related to hand hygiene, providing supportive care and education to mothers as well as surveillance-type midwifery practices.

## INTRODUCTION

Over the past ten years or so, the incidence and type of missed care across several nations have been reported but usually from a nursing perspective, and mostly then from the acute care sector. Kalisch^1^, in her pioneering work, not only defined missed care (as care left undone or omitted) but aligned the frequencies and types of missed nursing care to key reasons for their occurrences. Since that time, many missed care estimates have been applied to the acute hospital care^2^, aged care environments^3^, and community care^4^, but only minimally to the care of pregnant women, mothers/neonates and their families.

Core characteristics of midwifery care include optimizing normal biological, psychological, social and cultural processes of reproduction and early life, as well as timely prevention and management of complications^5^. These complications can include puerperal sepsis secondary to missed hand washing. WHO^6^ cautions that 30000 women and 400000 babies die every year worldwide from infections, and that midwives should pay particular attention to hand hygiene, especially during childbirth. This imperative, of course, becomes more urgent with the advent of COVID-19. Midwives’ support and empathy toward the mother are crucial ingredients of quality midwifery practice and can play a key role in creating a positive birth experience^7^. Therefore, midwives should provide care that is woman-centred, respectful of the woman’s individual circumstances and views, and serves to strengthen the women’s own capabilities to care for herself and her family^8,9^. However, there are factors during labour and birth that can inhibit full partnership between women and midwives. These include staffing levels, failure to apply evidence-based care, environmental constraints such as the room set-up^10^ and perinatal care.

Perinatal education is another core duty for midwives^9^ as it enables effective education on labour and childbirth for new families, and has the potential to reduce psychological distress and to maximize the involvement of partners^11^. Midwives are the ideal professionals to deliver perinatal education programmes because they are a stable point of contact, approachable and accessible to address and help resolve concerns by providing immediate care in a timely way to women and their babies^12^.

The aims of the study are to first quantify and scale the frequencies and types of missed Australian midwifery care, according to the differing complexity of the consensus estimates of Australian midwives. Additionally, the study seeks to identify if missed Australian midwifery care can be predicted. In other words, if the variances of missed midwifery care scores can be explained by modelling the numerous variables, which are proposed or hypothesized to be associated with missed Australian midwifery care.

## METHODS

### Design and setting

A non-experimental, descriptive method using two sections of Kalisch’s (2006) MISSCARE survey^1^ was used for this study of Australian midwives. The two subsections used included: 1) seeking information about the demographic features of the Australian midwife including his/her employment settings (12 questions); and 2) exploring the different elements of missed care using 17 self-reported Likert-type survey items.

### Participants

Midwife recruitment occurred through the membership of the South Australian, New South Wales, Victorian and Tasmanian branches of the Australian Nursing and Midwifery Federation (ANMF). Electronic invitations were extended to their membership using an inclusive link to the MISSCARE survey. Inclusion criteria were all ANMF members who were midwives and currently employed within the Australian public and private healthcare systems. A screening mechanism (an initial employment question) was used to exclude midwives who were not currently employed in their respective fields. Approval for the study was obtained from the relevant university Social and Behavioural Research Ethics Committees as well as the heads of the ANMF in each of the four Australian States participating in the survey. Participant midwives were advised that the research would provide information about when midwifery care was missed, and they were free to participate or not, at any time. To maximize midwife participation, recruitment invitations were later followed up by an advertisement in an electronic newsletter distributed to all ANMF members from the targeted Australian States. Complete responses to the survey were received from 386 Australian midwives.

### Instrument: the missed care midwifery scale

Our modified MISSCARE self-report survey contained 76 questions related to midwife demographics, including their healthcare setting type, and types and frequencies of missed care (omitted, postponed, or incomplete care) over three shifts of time: morning, evening, and overnight. Seventeen survey items were used to reflect midwifery care associated with monitoring mothers and babies (7 items) with the residual items exploring missed care related to their needs. A 5-point Likert scale was used to estimate the types and frequencies of missed midwifery care. As several factors used in this study were not directly observable (i.e. they were latent variables), the use of observable variables to describe them was required. Table 1 lists these latent and observed variables, all of which were derived from the midwives’ MISSCARE survey.

**Table 1:** Description of the survey items and variable names predicted to influence frequencies and types of missed midwifery care

*Midwives’ latent survey variables (LVs)*	*Name and description number of observed (indicator) variables*
1 Length of clinical experience	Years
2 Employment sector	Private hospital=1, Public hospital=2
3 Workplace location	City hospital=1, Regional/rural setting=2
4 Midwives’ highest education	Certificate=1, Registered Nurse=2, Diploma=3, Bachelor=4, Masters=5, Other=6
5 Australia as the country of initial midwifery registration	Yes=1, No=2
6 Employment status	Part-time=1, Full-time (>30 h)=2
7 Work roster preference	Say on current roster=1, change roster=2
8 Own health status	Excellent=1, Very good =2, Good=3, Fair=4, Poor=5
9 Adequacy of ward staffing	100% of time=1; 75%=2; 50%=3; 25%=4; 0%=5
10 Work satisfaction How satisfied are you in your current position? How satisfied are you with the level of teamwork in your unit/ward? Independent of your current position/job, how satisfied are you with being a midwife?	Very satisfied=1, Satisfied=2, Not satisfied=3, Very dissatisfied=4
11 Do you plan to leave your current position?	Yes=1, No=2
12 Do you use ‘rounding’ as part of your work?	Yes=1, No=2
13 Missed monitoring-based midwifery care	2. Undertaking vital signs as ordered 3. Monitoring intake & output 4. Full documentation of all necessary data 9. Bedside glucose monitoring as ordered 10. Focussed assessment according to patients’ condition 12. Responds to initiated call bell/light within 5 minutes 14. Assesses the effectiveness of medications
14 Missed treatment-related or needs based midwifery care	1. Administering medications within 30 minutes before or after scheduled time 5. Patient education about illness, test & diagnostic studies 6. Emotional support to patient and/or family 7. Hand hygiene/washing 8. Patient discharge planning & education 11. I/V & central line site care & assessment 13. PRN medication requests acted upon within 15 minutes 15. Skin/wound care 16. Patient bathing/skin care 17. Mouth care
15 All missed midwifery care	Total of all missed care scores^a^

a Scale: 1=never missed, 2=rarely missed, 3=occasionally missed, 4=frequently missed, 5=always missed.

#### Constructing the hypothetical model predicting Australian missed midwifery care

**Figure 1:**
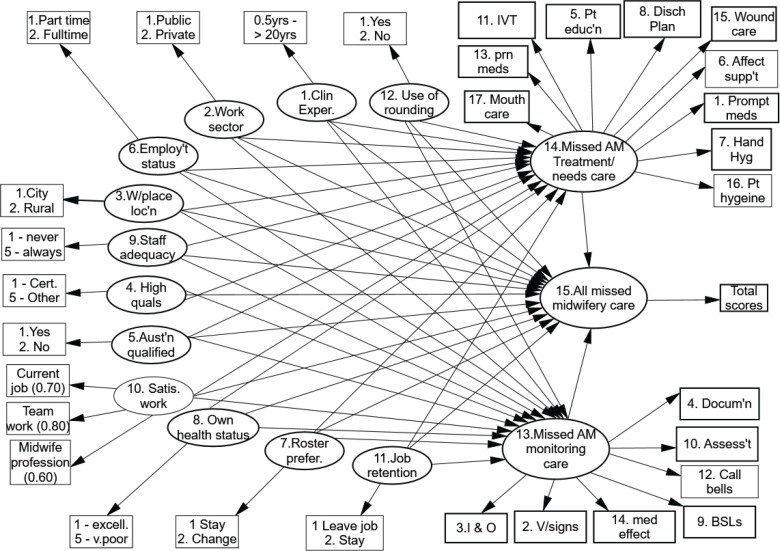
The hypothetical model accounting for the frequencies and types of missed midwifery care: morning shift

Constructing the hypothetical model predicting Australian missed midwifery care To find out which factors have an established and significant effect on the frequencies of missed midwifery care, a path model or an arrow scheme is used and diagrammatically presented in Figure 1. It is proposed/hypothesized that all variables (numbered 1 to 14 and called ‘latent variables’ (LV) will have some effect on the total frequencies of missed midwifery care, be it the type of workplace the midwife is employed in, staffing adequacy and job retention, for example. It is further proposed that each of the midwives’ demographic factors will also influence the frequencies and types of missed surveillance or monitoring type midwifery care.

### Statistical analysis

The Likert-type scale used in this current study involves ordinal measures that cannot be analysed using traditional methods of statistical analysis^13^. Instead, Rasch analysis was employed to measure the reliability indices for both the midwives’ responses and for the instrument’s survey items findings^14^. Rasch modelling, specifically the Winsteps program^15^ was used to determine the validity of all the participants’ responses. Structural equation modelling (PLS-SEM) was used to identify and highlight the significance of the relationships between variables that were thought to influence the incidences and types of missed midwifery care^16^. This form of multivariate analysis (PLS-SEM version 3) sets out to explain as much of the variance in the scores of the midwives’ responses when rating missed midwifery care. It is in this way that the antecedent factors (or variables) can be predicted, modelled, and understood in a robust fashion^17^. PLS-SEM is particularly useful for analysing complex data arising from clinical settings that would otherwise make traditional analysis (such as regression) difficult to use^18^.

## RESULTS

### Reliability of the missed care survey items

Cronbach’s alpha was not used during analysis due to its inability to determine if the missed midwifery care survey items are all measuring the same underlying construct^13^ . Instead, Rasch analysis was used as it is able to simultaneously identify if the midwives’ response patterns to the survey items was overly repetitive or if they avoided using certain scale categories^19^. Rasch analysis also uses two reliability indices: Person Separation Index (PSI), and Item Separation Index (ISI). These indices analyse participant and item response patterns independently of the reliability of the survey items, which Cronbach’s alpha is not able to do. To demonstrate acceptable reliability, the PSI and ISI indices need to be above 2.0. Our survey values were PSI=3.16 (reliability=0.94) and ISI=8.98 (reliability=0.99), which are very acceptable reliability indices^14^. Missing data were minimal, but to maximize data reliability, missing data were managed by using the maximum imputation method.

Table 2 highlights the patterns of demographic data outcomes from the surveyed Australian midwives. Most candidates hold an initial Australian derived midwifery registration, at post-graduate level and are well experienced in this field (16 to 20 years plus). Most midwives are employed on a part-time basis and employed in city-based public hospitals.

**Table 2:** Distribution of Australian midwives’ demographic data (N=386)

*Demographic variable*	*n*	*%*
**Length of clinical experience** (years)		
<1	10	3
≤2	25	7
3–4	27	7
5–6	23	6
7–8	20	5
9–10	17	4
11–15	42	11
16–20	36	9
>20	185	48
**Employment sector**		
Public sector	312	81
Private hospitals	74	19
**Workplace location**		
City based	178	46
Rural/Regionally based	122	31
Not specified	86	23
**Maternity staffs’ highest education**		
Enrolled Nurse	7	2
Registered General Nurse	67	17
Bachelor Degree in Nursing/Midwifery	48	12
Graduate Diploma in Nursing/Midwifery	145	38
Master’s Degree	78	21
PhD	5	1
Other	36	9
**Australia as the country of initial midwifery registration**		
Yes	339	88
No	47	12
**Employment status**		
Part-time	274	71
Full-time	112	29

**Figure 2:**
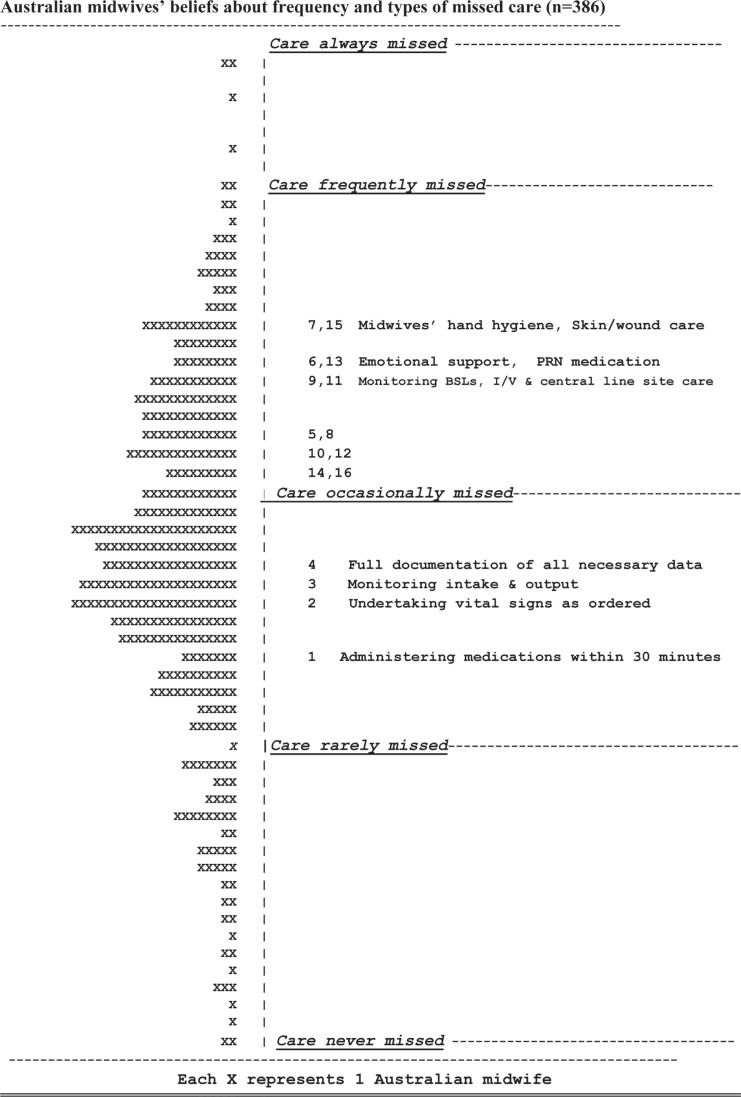
Distribution of missed midwifery care based on Australian midwives’ consensus

Figure 2 demonstrates the frequency and types of day shift missed midwifery activities, as shown by the consensus of Australian midwives.

### Quantifying missed midwifery care

About 1% of the midwifery group believed that midwifery care was always missed, with another 45% of midwives estimating care to be frequently missed, and 41% of midwives indicating care was missed occasionally. Midwifery care was seen to be never missed by the remaining 13% of participants.

Figure 1 additionally demonstrates a hierarchical, interval scale of the frequencies and types of missed midwifery care providing valuable insights into the capacity of the participant midwife to care for mothers and their families. Survey items 7 and 15 are located towards the top of the missed care scale (performing hand hygiene, undertaking skin/wound care, respectively) and are the care activities most frequently missed by midwifery staff. As items 7 and 15 are co-located at the same level on the missed care scale, this confirms that both are missed as frequently. Providing PRN medication promptly (item 13) and providing the mother and her family with emotional support (item 6) are only marginally to be missed less frequently. Monitoring the mother’s blood sugar levels and parenteral lines are cited as being missed frequently. Items 5 and 8 refer to educating the mother about any diagnostic test she may be encountering and upon discharge. It is noted that these two aspects of care are rated as being missed occasionally to frequently. Item 10 refers to the frequency in which the midwife evaluates the condition and care of the mother in her care, and this aspect is missed as frequently as is the promptness in responding to call bells (item 12). Evaluation of any medication as to its effectiveness when given to the mother (item 14) is missed as frequently as midwife-initiated bathing and skincare afforded to the mother.

Conversely, at the lower end of the interval scale in Figure 2, are those aspects of midwifery care missed the least often. In hierarchical order, they include administering prescribed medications promptly (item 1) taking observations (item 2), monitoring fluids (item 3) and documenting care given (item 4). These last aspects of care are thought to be missed occasionally.

### Predicting different variables’ influence on missed midwifery care

**Figure 3:**
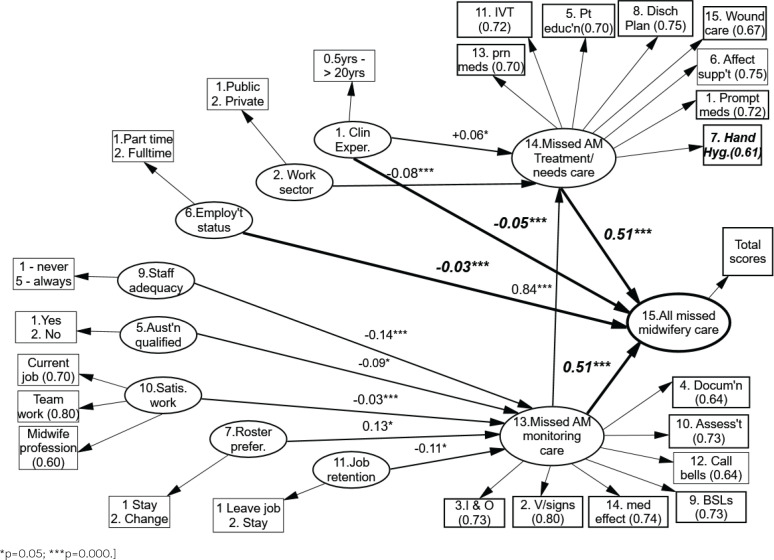
Final model showing variables that predict types and frequencies of missed Australian midwifery care: morning shift

Figure 3 shows the final outcomes of the hypothetical model as tested against the Australian midwives’ beliefs about the care that they had missed. Of the 14 variables that were believed to influence the frequencies of missed midwifery care, ten latent variables (LV) remain as statistically significant variables for predicting overall missed midwifery care. Workplace location (LV3), staff qualification (LV4), staff health status (LV8) and the use of rounding (LV12) were not statistically significant at predicting missed midwifery care, so they were subsequently removed from the final missed midwifery care model. Figure 3 shows both the direct and indirect effects of the variables on Australian missed midwifery care.

It can be seen from Figure 3 that four variables have direct influence on all missed Australian midwifery care scores (as shown by the four bold arrows arriving at LV15). Variances in the total missed midwifery care scores are equally divided by the influences of two major variables: the frequencies needs-based missed care (LV14), and surveillance or monitoring-type missed midwifery care (LV13). Importantly, missed opportunities in surveillance type midwifery care (LV13) additionally exert a direct effect on the frequency of omitted needs-based midwifery care (LV14). The years of midwifery clinical experience (LV1) as a factor in accounting for missed midwifery care is significant in two related ways. Staff with longer years of clinical experience indicate that needs-based midwifery care, specifically, is most likely to be omitted. Still, it is the more novice midwives who suggest that all incidences of midwifery care are missed overall. All aspects of missed midwifery care are more likely to be reported as being missed by full-time staff (LV6) compared to midwives working under 30 hours per week. Needs-based midwifery care (LV14) is reported to be more frequently missed according to public hospital midwifery staff compared to their colleagues working within the private sector (LV2). Missed surveillance type midwifery care is in turn directly impacted on by perceived adequacy of staff on the midwifery wards (LV9), where perceived insufficient staff numbers account for more missed care. This is particularly so for midwives who were qualified in Australia (LV5) and by those midwives who are dissatisfied with the level of teamwork and with their current job overall (LV10). Lastly, midwives who are both happy with their roster arrangements and who do not indicate an intention to leave their current work (LVs 7 and 11) are accounting for more missed care than midwives who wish to leave their work and would prefer a roster change to their current arrangements.

## DISCUSSION

Findings reveal that the most prevalent aspects of missed care in the Australian midwifery setting are midwives’ hand hygiene, supportive care, perinatal education and surveillance type midwifery practices. These omissions are discussed in the context of the different demographic factors that have demonstrated a significant effect on missed care.

### Midwives’ hand hygiene

Effective and timely hand hygiene is the important intervention against hospital acquired infections and remains a barrier to delivering high-quality and safe care in health facilities. In maternity settings, inadequate hand hygiene practices during perinatal care negatively affect the health of both mothers and neonates. This is consistent with WHO^6,20^, which documented that low hand hygiene protocol is an acute stumbling block to achieving quality of care. Thus, it is crucial that all midwives wash their hands before and after midwifery care and after being exposed to bodily fluids. This study has shown that it is the more experienced midwives working in the public sector who are least likely to miss hand hygiene compared to their younger counterparts and those employed in the private maternity care environments. However, up to 70% of health workers do not adhere to recommended hand hygiene practices, according to WHO^20^. One study indicates that the rates of hand washing were low amongst doctors and suggests that there is a need for regular education of doctors and provision of better facilities for hand washing^21^. This is relevant for novice midwives and private hospital staff too, as was shown in this study, where the younger and privately employed midwives were more likely to miss hand hygiene. Poor compliance with hand hygiene protocols has historically been strongly associated with structural barriers, including overcrowding, high patient loads and understaffing^22^, limited time^23^, and inadequate infrastructure^24^. However, in one observational study conducted in six midwifery settings in Nigeria, five women were observed from the onset of labour through to delivery of the placenta. Hand hygiene infection risk was estimated for all procedures requiring aseptic technique compared with adherence to proper hand hygiene protocol and potential recontamination events. Data analysis identifies that adherence to proper hygiene protocol was observed more in the morning compared to afternoon and night shifts^25^. The hand hygiene of midwives is the cornerstone of quality care and hand hygiene protocols are a critical component of infection prevention strategies and the minimization of maternal and neonatal mortality rates. Additionally, in the current pandemic of COVID-19, midwives are facing enormous challenges, proper hand washing with soap and water for at least 20 seconds is vital for effective infection prevention and control measures. Hand hygiene is crucial for ensuring that health facilities do not become hubs of COVID-19 transmission and reducing healthcare-associated infections from other pathogens. Midwives must be provided with the resources and training required to implement good hand hygiene practices to respond to the pandemic and to maintain essential perinatal services safely^26^.

### Perinatal education

The transition to parenthood is a potentially vulnerable time for a mother’s mental health, as approximately 9–21% of Australian women experience depression and/or anxiety at this time^27^. Furthermore, the absence of adequate maternal social support further exacerbates the risk of developing depression^28^. This is particularly true for women who have insufficient partner support or are exposed to violence, who come from a non-English speaking background, and have a low socio-economic status^29^. During the more immediate period of the pregnancy, if the mother’s level of anxiety is significant or if she has feelings of being unsupported, this in turn can lead to stress during labor, with a concomitant release of excessive beta-endorphins which in turn inhibit oxytocin release. Additionally, the secretion of epinephrine and norepinephrine (flight or fight hormones) can also simultaneously inhibit oxytocin, making both labor and the birth longer and more difficult^30,31^. While the biggest risk factor for the development of post-natal depression is being depressed during the pre-natal period, midwives need to be ready to assess changes in mood during all stages of pregnancy. Being able to differentiate between alterations to the mothers’ affective state, and its significance for the development of depression, is dependent on the midwives’ knowledge of the different stages of pregnancy and severity of affective changes. Emotional lability and tearfulness are typical for most mothers in the immediate postpartum period requiring staff re-assurance, but its prolongation and deeper intensity of mood may herald the onset of postnatal depression and a more dynamic response^32^. While one of the key features of both ante and post-natal depression is its congruence between the mothers’ statements of sadness and their physical expression of depressed behaviour, the midwives’ health assessment skills and an improved ability to recognize anxiety or depression can be enhanced with the use of different tools that seek to identify the mothers’ underlying affect, such as the Edinburgh Post Natal Depression Scale^33^ and the Mood Disorders Scale^34^.

Current literature suggests that there may be also no link between the nature and degree of support offered to mothers by their midwives^7^. These authors suggest that while midwives themselves do not always agree about what is actually involved in providing mothers with emotional support, generally midwives see their role of providing emotional support as essentially focussing on assisting with a normal birthing experience and increasing the mother’s confidence in care of her baby. Conversely, the mother’s pivotal expectations of a midwife are for someone who listens to them, shows respect, acceptance, and provides clear information^35,36^. The Australian Nurses and Midwifery Board^37^, the Australian College of Midwives^38^ and the ICM^9^ have similar expectations of both parties cited here, suggesting that midwives are required to be truly present with a woman in labor, to be empathic with the mother as she undergoes the deep sensations and emotions of birthing, while administering appropriate care, kindness and compassion. A key component to emphasize here is the targeted development of the midwives’ use of empathy as a mechanism for providing support to the mothers. Midwives can better demonstrate empathy through the therapeutic use of self; by being present not just physically, by providing continuity of care, but by being present psychologically too. Empathy can be facilitated when the midwife uses appropriate self-disclosure to facilitate the relationship through trust development and by acknowledging the circle of care that exists by being inclusive with the mother’s family. One study suggests that meeting the emotional needs in another person necessitates a healthcare professional’s own emotional involvement, which has the potential to cause emotional stress in healthcare professionals^39^. Increased trust and support have been found in the antenatal continuity of care to be fundamental to a midwife’s role and have been seen to decrease birth trauma and fear of childbirth^40^, but also affect the post-natal outcomes of both the mother and child^41^. Whilst many venues have policies on post-birth care plans, e.g. education of the woman and her family, there appear to be minimal practice examples^42^. However, in many countries, midwives are offered antenatal education without taking into consideration of the woman’s personal context and it is limited to the two months before childbirth and with an emphasis on the provision of information during pregnancy^12^.

Significant decreases in post-natal length of stays in hospital present limited opportunities to provide post-natal education and undertake discharge planning^43^. Decreased post-natal time, increasing staffing pressures and emotionally stressed midwives contribute to high attrition and to missed care^44^. This situation is also exacerbated when midwives are not satisfied with their work, team roles or roster preference, hastening a desire to leave their current employment and mounting further pressure on staffing levels. This study has also shown that missed care, as it relates to post-natal education and discharge planning, is in turn influenced by the frequency of other types of missed midwifery care such as monitoring care of mothers and babies. What in effect happens is that a cascade of missed maternity care occurs; when there are multiple deficits in monitoring-based care, needs-based care is affected also, including post-natal education and discharge planning^45^. Midwives need to offer early and realistic information about parenting skills and more support and help for the postnatal period. Creating a parenthood education program that suits all participants, with their various expectations and needs^12^, is a major challenge.

### Surveillance or monitoring-type missed care

This type of missed care includes midwives failing to undertake ongoing re-assessment (or evaluation) of the mother and responding to call bells, which were noted to be issues of frequently missed midwifery care. This study also demonstrated that midwives that had not obtained their qualifications locally, were not happy with their job and current roster were more likely to miss this cluster of midwifery care. One British study of mothers who presented with excessive postpartum blood loss, showed that that this arose because of the midwives’ lack of assessment or evaluation of a mother experiencing perineal and vaginal tears secondary to a shoulder dystocia^45^. Another study, in Australia, indicated that midwives were indeed so frustrated with the disruption in the continuity of the care, due the continual call-bell use, that they had redesigned their work so call bells were to be used for emergency use only^46^.

### Limitations

While this study captured a significant number of consensus estimates from Australian midwives, its findings are not generalizable because they may not be reflective of the wider Australian midwifery population. The findings are, however, persuasive of missed midwifery practices, as the study was undertaken when there was likely to be a maximum complement of midwifery staff in the clinical area to provide care, i.e. during the day shift and where the variance of missed care may increase when there are reduced number of staff over different shifts.

## CONCLUSIONS

Decreasing maternity-based missed care involves not only working with current midwives but also changing the mindset of future graduates of midwifery. Increased focus on undergraduate midwifery educational activities such as strengthening the importance of care continuity, by reflective writing and advancing midwifery education including perinatal health experience, enables students to develop occupational competence^47,48^ with the potential to a decrease in missed care events in the future. The findings may be useful to advise midwifery curricula and inform the theoretical and skills-based practical education of midwifery students.

## References

[1] Kalisch B (2006). Missed nursing care: a qualitative study. J Nurs Care Qual.

[2] Blackman I, Henderson J, Willis E (2015). Factors influencing why nursing care is missed. J Clin Nurs.

[3] Blackman I, Henderson J, Weger K, Willis E (2019). The Casual Links associated with Missed Residential Aged Care. J Nurs Manag.

[4] Phelan A, McCarthy S, Adams E (2018). Examining missed care in community nursing: A cross section survey design. J Adv Nurs.

[5] Horton R, Astudillo O The power of midwifery. Lancet.

[6] World Health Organization Clean Care is Safer Care: WHO guidelines on hand hygiene in health care.

[7] Gair S, Moloney S (2015). Empathy and spiritual care in midwifery practice: Women contributing to women's enhanced birth experiences. Women Birth.

[8] Bradfield Z, Hauck Y, Duggan R, Kelly M (2019). Midwives' perceptions of being 'with woman': a phenomenological study. BMC Pregnancy Childbirth.

[9] Australian College of Midwives Philosophy & Values. https://www.midwives.org.au/midwifery-philosophy-values.

[10] Seibold C, Miller M, Hall J (1999). Midwives and women in partnership: the ideal and the real. Aust J Adv Nurs.

[11] Roch G, Da Silva R, de Montigny G (2018). Impacts of online and group perinatal education: a mixed methods study protocol for the optimization of perinatal health services. BMC Health Serv Res.

[12] Artieta-Pinedo I, Paz-Pascual C, Grandes G, Espinota M (2017). Framework for the establishment of a feasible, tailored, and effective perinatal education programme. BMC Pregnancy Childbirth.

[13] Sitjsma K (2009). On the use, the misuse, and the very limited usefulness of Cronbach's Alpha. Psychometrika.

[14] Boone W, Staver JR, Yale M (2014). Rasch analysis in Human Sciences.

[15] Linacre JM (2012). Rasch measurement software [computer program]. Version 4.5.4.

[16] Hansmans KW, Ringle C (2004). Smart PLS Manual. Version 3.0.

[17] Hair J, Hult G, Ringle C, Sarstedt M (2016). A Primer on Partial Least Squares Structural Equation Modelling.

[18] Haenlein M, Kaplan AM (2004). A beginner's guide to partial least squares (PLS) analysis. Understanding Statistics.

[19] Bond T, Fox C (2015). Applying the Rasch Model: Fundamental Measurement in the Human Sciences.

[20] World Health Organisation Clean Care is Safer Care: The evidence for clean hands.

[21] Alex-Hart B, Opara P (2011). Handwashing practices amongst health workers in a teaching hospital. Am J Infect Dis.

[22] Kuzu N, Ozer F, Aydemir S, Zencir M, Yalçın AN (2005). Compliance with hand hygiene and glove use in a university-affiliated hospital. Infect Control Hosp Epidemiol.

[23] Pittet D (2003). Hand hygiene: Improved standards and practice for hospital care. Curr Opin Infect Dis.

[24] Zingg W, Holmes A, Dettenkofer M (2015). Hospital organization, management, and structure for prevention of health-care-associated infection: A systematic review and expert consensus. Lancet Infect Dis.

[25] Buxton H, Flynn E, Oluyinka O (2019). Hygiene During Childbirth: An Observational Study to Understand Infection Risk in Healthcare Facilities in Kogi and Ebonyi States, Nigeria. Int J Environ Res Public Health.

[26] Singh K (2020). Promote hand hygiene to save lives and combat COVID-19.

[27] McLeish J, Redshaw M (2017). Mothers' accounts of the impact on emotional wellbeing of organized peer support in pregnancy and early parenthood: a qualitative study. BMC Pregnancy Childbirth.

[28] Littleton H, Breitkopf C, Berenson A (2007). Correlates of anxiety symptoms during pregnancy and association with perinatal outcomes: a meta-analysis. Am J Obstet Gynecol.

[29] Ogbo F, Eastwood J, Hendry J, Agho B (2018). Determinants of antenatal depression and postnatal depression in Australia. BMC Psychiatry.

[30] Buckley C (2015). Hormonal Physiology of Childbirth: Evidence and Implications for Women, Babies and Maternity Care.

[31] Samsone A Connection and Empathy. Midwifery Today.

[32] Melville J, Gavin A, Guo Y, Fan M, Katon W (2010). Depressive Disorders During Pregnancy: Prevalence and Risk Factors in a Large Urban Sample. Obstet Gynecol.

[33] Cox J, Holden J, Sagovsky R (1987). Detection of postnatal depression. Development of the 10-item Edinburgh Postnatal Depression Scale. Br J Psychiatry.

[34] Hirschfield R (2002). The Mood Disorders Scale: a simple patient-rated screening instrument for bipolar disorders. Prim Care Companion J Clin Psychiatry.

[35] Barker S (2011). Midwives' Emotional Care of Women becoming Mothers.

[36] Priel B, Besser A (2002). Perceptions of early relationships during the transition to motherhood: the mediating role of social support. J Obstet Gynecol Neonatal Nurs.

[37] Australian Nurses and Midwifery Board of Australia Code of Conduct for Midwives.

[38] International Confederation of Midwives Essential Competencies for Midwifery Practice: 2019 Update.

[39] Thomas R, Wilson J (2004). Issues and controversies in the understanding and diagnosis of compassion fatigue, vicarious traumatization and secondary traumatic stress disorder. Int J Emerg Ment Health.

[40] Hildingsson I, Karlstrom A, Rubertsson C, Haines H (2019). Women with fear of childbirth might benefit from having a known midwife during labour. Women Birth.

[41] D'haenens F, Van Rompaey B, Swinnen T, Tinne Dilles E, Beeckman K The effects of continuity of care on the health of mother and child in the postnatal period: a systematic review. Eur J Public Health.

[42] Crowther S, MacIver E, Lau A (2019). Policy, evidence and practice for post-birth care plans: a scoping review. BMC Pregnancy Childbirth.

[43] Goodwin L, Taylor B, Kobab F, Kenyon S (2018). Postnatal care in the context of decreasing length of stay in hospital after birth: The perspectives of community midwives. Midwifery.

[44] Cramer E, Hunter B (2019). Relationships between working conditions and emotional wellbeing in midwives. Women Birth.

[45] Healey S, Humphreys E, Kennedy C (2017). A Qualitative Exploration of How Midwives' and Obstetricians' Perception of Risk Affects Care Practices for Low-Risk Women and Normal Birth. Women Birth.

[46] Morrow J, Mc Lachlan H, Forster D, Davey M, Newton M (2013). Redesigning Postnatal Care: Exploring the Views and Experiences of Midwives. Midwifery.

[47] Hezelgrave N, Abbott D, Shennan A (2015). Challenging Concepts in Obstetrics and Gynaecology: cases with Expert Commentary.

[48] Sweet L, Bass J, Graham K, Billett S, Newton J, Rogers G, Noble C (2019). The Continuity of Care Experience and Reflective Writing: Enhancing Post-Practicum Learning for Midwifery Students. Augmenting Health and Social Care Students' Clinical Learning Experiences. Professional and Practice-based Learning.

